# Bushfire smoke is pro-inflammatory and suppresses macrophage phagocytic function

**DOI:** 10.1038/s41598-018-31459-6

**Published:** 2018-09-07

**Authors:** Rhys Hamon, Hai B. Tran, Eugene Roscioli, Miranda Ween, Hubertus Jersmann, Sandra Hodge

**Affiliations:** 1 Chronic Inflammatory Lung Disease Research Laboratory, Department of Thoracic Medicine, Royal Adelaide Hospital, Adelaide, Australia; 2 Department of Medicine, University of Adelaide, Adelaide, Australia

## Abstract

Bushfires are increasing in frequency and severity worldwide. Bushfire smoke contains organic/inorganic compounds including aldehydes and acrolein. We described suppressive effects of tobacco smoke on the phagocytic capacity of airway macrophages, linked to secondary necrosis of uncleared apoptotic epithelial cells, persistence of *non-typeable H. influenzae* (NTHi), and inflammation. We hypothesised that bushfire smoke extract (BFSE) would similarly impair macrophage function. THP-1 or monocyte-derived macrophages (MDM) were exposed to 1–10% BFSE prepared from foliage of 5 common Australian native plants (genus Acacia or Eucalyptus), or 10% cigarette smoke extract (CSE). Phagocytic recognition receptors were measured by flow cytometry; pro-inflammatory cytokines and caspase 1 by immunofluorescence or cytometric bead array; viability by LDH assay; and capsase-3/PARP by western blot. BFSE significantly decreased phagocytosis of apoptotic cells or NTHi by both THP-1 macrophages and MDM vs air control, consistent with the effects of CSE. BFSE significantly decreased MDM expression of CD36, CD44, SR-A1, CD206 and TLR-2 and increased active IL-1β, caspase-1 and secreted IL-8. BFSE dose-dependently decreased THP-1 macrophage viability (5-fold increase in LDH at 10%) and significantly increased active caspase-3. BFSE impairs macrophage function to a similar extent as CSE, highlighting the need for further research, especially in patients with pre-existing lung disease.

## Introduction

Bushfire severity and frequency is increasing worldwide and further increases are predicted. One study comparing the incidence of major fires from 1970 to 2012 with precipitation and land-surface data confirmed an increase in bushfire activity associated with prolonged droughts^1^. Similar trends have been observed in Australia where average temperatures have risen by 0.9 °C since 1910 and average winter rainfall has decreased by 15–17% in southern Australia^2^. The increased frequency and severity of bushfires taken together with a predicted yearly occurrence of severe fire danger rated days, increasing by up to 300% by 2050^3^, presents a greater risk of smoke exposure to the population. The United States urban interface with wildland areas increased by 52% from 1990 to 2000^4^. This increase in bushfire frequency, coupled with global populations encroaching on forest areas, means that large communities are increasingly required to co-exist with both unplanned and prescribed wildfires^5^.

The interface of urban epidemiological studies have found significant associations between bushfire smoke exposure and both emergency department presentations^6,7^ and hospital admissions^8,9^ for respiratory morbidities. An Australian based study conducted by Martin *et al*.^9,10^ reported increased hospital admissions for pre-existing respiratory morbidities in association with bushfire events; 13% for chronic obstructive pulmonary disease (COPD) and 12% for asthma. This is supported by a California based study where admissions were increased by 4.8% for COPD and 6.8% for asthma^8^. Furthermore, healthy subjects exposed to higher levels of particulate matter (PM) during a Southeast Asian forest fire event exhibited increased systemic inflammatory responses, including increased band neutrophil counts^11^ and elevated cytokines in peripheral blood^12^. Respirable particulate matter from bushfire events in California was reported to elicit oxidative stress and inflammatory responses. Human bronchial epithelial cells exposed to PM *in vitro* expressed significantly more IL-8 and IL-1β^13^ while bronchoalveolar lavage from exposed mice contained higher levels of MIP-1α and IP-10^14^.

Bushfire smoke contains a range of organic and inorganic components such as respirable particulate matter, aldehydes, acrolein, and carbon monoxide^15^. Very little is known about its effects on airway epithelial cells and alveolar macrophages that represent the frontline immunological barrier to potentially toxic particles. One study showed that California bushfire particulate matter alone resulted in reduced number of lung macrophages in mice^16^ and another found increased macrophage cell death *in vitro*^17^. In contrast, the negative effects of tobacco smoke on these cell types has been well-described. We and others reported that cigarette smoke caused increased apoptosis of bronchial epithelial cells with an associated defect in the capacity of neighbouring macrophages to phagocytose these apoptotic cells (efferocytosis)^18–21^. Similar defects were noted in patients with chronic lung diseases including COPD, severe asthma, and childhood non-CF bronchiectasis^22–24^. This defect was associated with secondary necrosis of the uncleared material, potentially leading to release of toxic cell contents, perpetuating inflammation and further tissue damage^19,25^. We also found that cigarette smoke exposure inhibited the capacity of alveolar macrophages to phagocytose bacteria, including non-typeable *H. influenzae* (NTHi), a common coloniser of the airway in chronic lung diseases that is associated with significant morbidity. To our knowledge, the effects of bushfire smoke on macrophage phagocytic function has not been previously described. We thus hypothesised that exposure to a bushfire smoke extract (BFSE) causes defects in macrophage function that parallel our findings with cigarette smoke extract (CSE).

## Results

### BFSE suppressed macrophage phagocytic capacity

**Figure 1:**
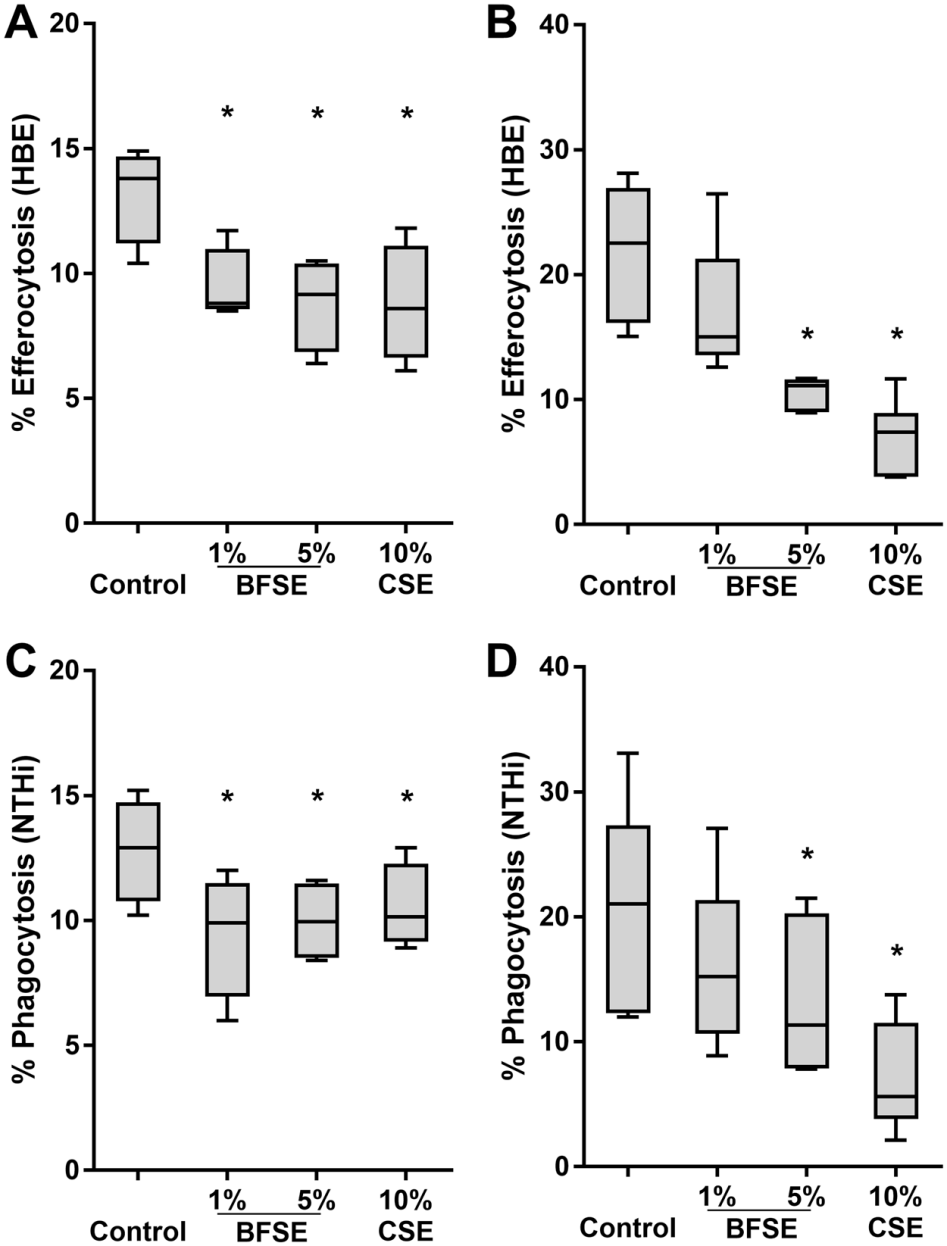
BFSE suppressed macrophage phagocytic capacity. THP-1 macrophage and MDM cells exposed to air control, 1% or 5% BFSE, or 10% CSE for 24 h were cultured with apoptotic epithelial cells and NTHi. Efferocytosis of apoptotic epithelial cells by THP-1 macrophage (**A**) and MDM (**B**) and phagocytosis of NTHi by THP-1 macrophage (**C**) and MDM (**D**) were assessed by flow cytometry and measured as the percentage of cells positive for internalisation of targets (THP-1 n = 4, MDM n = 6). *p < 0.05.

The capacity of THP-1 macrophages and MDM to efferocytose apoptotic HBE cells was significantly decreased after 24 h of exposure to BFSE compared to the air treated control. THP-1 macrophage efferocytosis was significantly (p = 0.012) decreased from 13.8% by control cells to 9.5% and 8.8% with 1% and 5% BFSE, respectively (Fig. 1A). MDM efferocytic capacity was decreased from 21.9% to 17.1% and 10.6% (p = 0.031), for 1% and 5% BFSE, respectively (Fig. 1B). For efferocytosis, MFI was not significantly changed by any treatment (Supplementary Fig. 1).

The capacity of THP-1 macrophages and MDM to phagocytose NTHi was also significantly reduced following 24 h exposure to BFSE. THP-1 macrophage exposure to 1% or 5% BFSE decreased phagocytosis from 12.9% to 9.9% and 9.9% respectively (p = 0.012, Fig. 1C). Exposure of MDM to 1% or 5% BFSE resulted in a decrease in phagocytosis from 20.8% to 16.2% and 13.3% (p = 0.031), respectively (Fig. 1D). Observed decreases in efferocytic and phagocytic function after 24 h exposure to 5% BFSE were to a similar extent as with 10% CSE treatment for 24 h for both THP-1 macrophage and MDM. For phagocytosis, a significant reduction in MFI was noted in the presence of both 1% and 5% BFSE (Supplementary Fig 1).

### BFSE alters phagocytic recognition receptors

**Figure 2:**
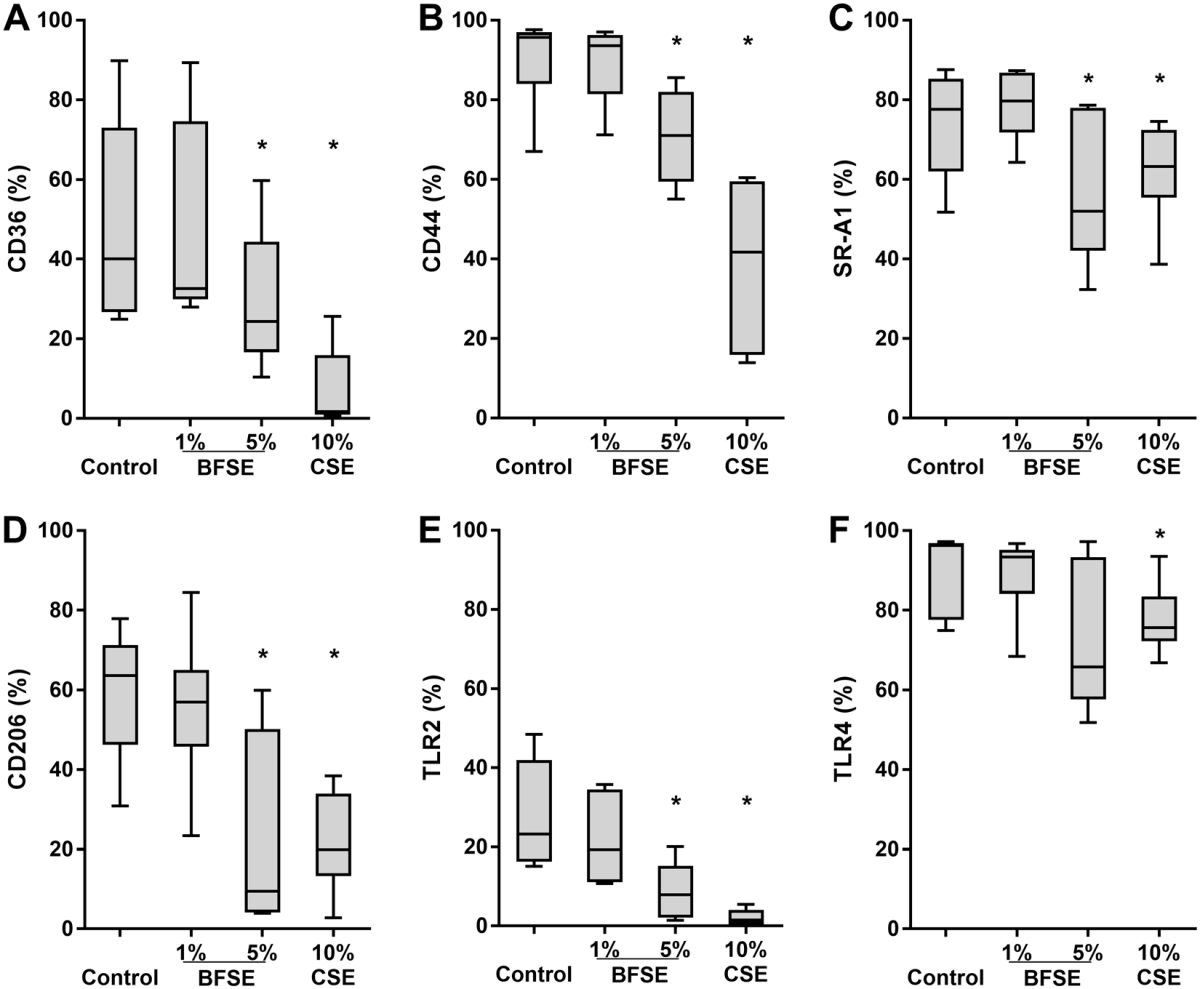
BFSE alters phagocytic recognition receptors. MDM were exposure to air control, 1% or 5% BFSE or 10% CSE for 24 h. Cell surface markers: CD36 (**A**), CD44 (**B**), SR-A1 (**C**), CD206 (**D**), TLR-2 (**E**) and TLR-4 (**F**) were detected by immunofluorescence on a FACScanto II flow cytometer and expressed as % positive cells compared to negative controls. (n = 5) *p < 0.05.

Effective phagocytosis of bacteria or efferocytosis by macrophages is dependent on the expression of surface recognition receptors. To investigate the effects of BFSE or cigarette smoke on the percentage of cells expressing thrombospondin receptor (CD36), hyaluronan receptor (CD44), scavenger receptor (SR)-A1 (CD204), mannose receptor (CD206) and toll-like receptors (TLR) −2/4, MDM were exposed to 1% or 5% BFSE and 10% CSE for 24 h, then receptors measured using flow cytometry. There were no significant effects of 1% BFSE on any of the receptors tested (Fig. 2). Both 5% BFSE and 10% CSE induced a significant decrease in the percentage of cells expressing CD36, CD44, CD204, CD206 and TLR-2 (Fig. 2). There was also a decrease in the MFI of staining, (Supplementary Fig. 2), indicating that the amount of receptors being expressed is being reduced, alongside the % of cells expressing the receptors. Expression of TLR-4 decreased after exposure to 5% BFSE, but was unchanged by CSE (Fig. 2).

### BFSE increases intracellular pro-inflammatory mediators

**Figure 3:**
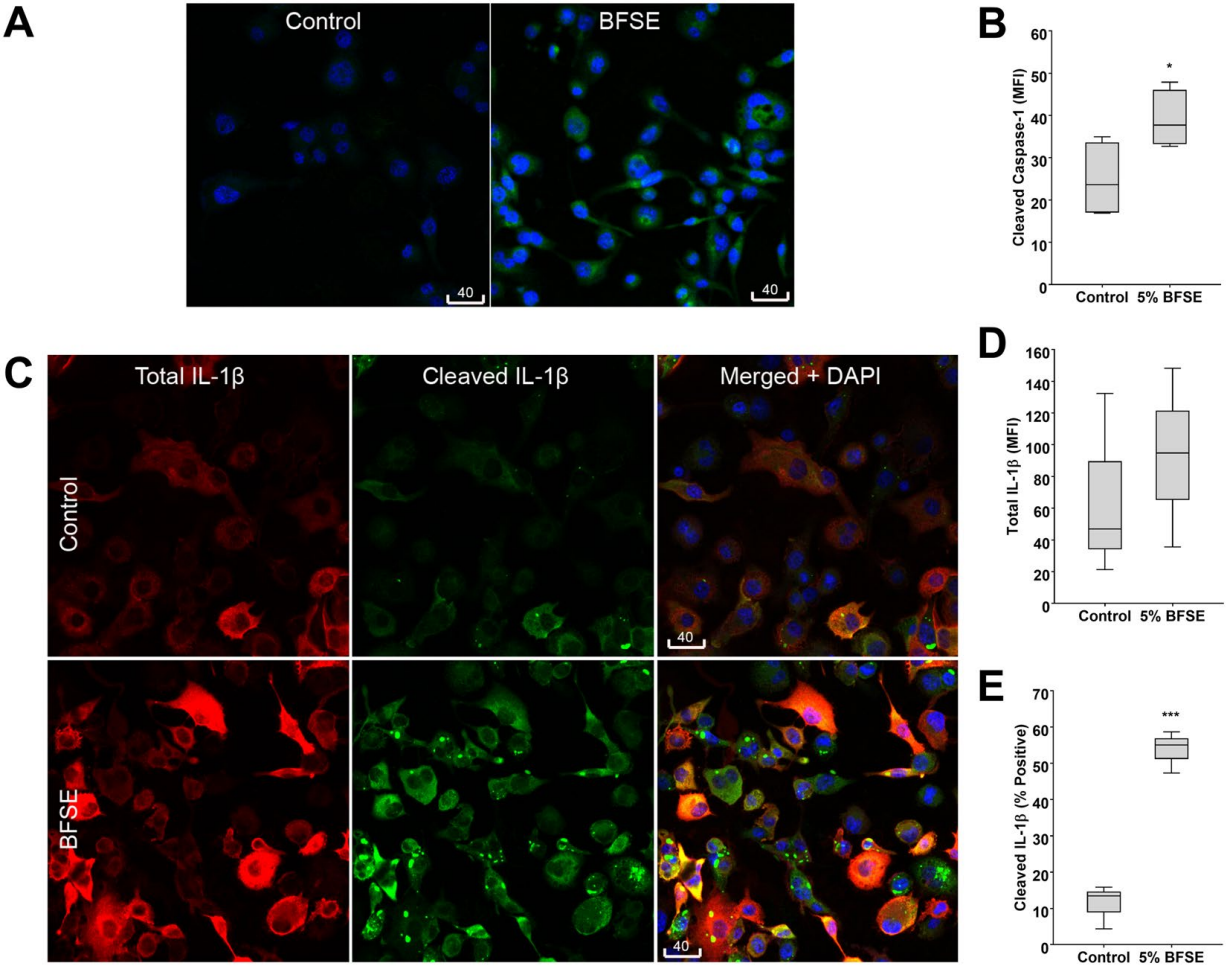
BFSE increases intracellular pro-inflammatory mediators. THP-1 macrophage cells were exposure to air control or 5% BFSE for 24 h. Active Caspase-1 (**A**) and; total IL-1β and active IL-1β (**C)** were detected by immunofluorescence. Confocal images were quantitatively measured for MFI of active Capase-1 (**B**) and total IL-1β (**D**), or the percentage of cells containing bright particulate active IL-1β (**E**). (n = 4) *p < 0.05 ***p < 0.001.

The inflammatory response initiator cleaved caspase-1 was detected in the air treated control THP-1 macrophage but to a greater extent in cells exposed to 5% BFSE for 24 h (Fig. 3A). The MFI of intracellular cleaved caspase-1 in 5% BFSE treated cells was significantly (p = 0.047) higher than air treated control, 39.0 vs 24.7 (Fig. 3B).

Increases in both precursor and cleaved IL-1β immunofluorescence were observed in 5% BFSE-treated THP-1 macrophages compared to air control (Fig. 3C). Detection of IL-1β using an antibody directed to amino acids 117–269, present in both the active and precursor IL-1β, revealed a homogenous cytoplasmic distribution which was non-significantly increased, MFI: 99.0 vs 67.2, 5% BFSE vs control (Fig. 3D). Cleaved IL-1β specific detection using an antibody reacting with the Asp 116 neoepitope on the IL-1β cleaved end but not cross-reacting with the precursor cytokine, revealed both homogenous cytoplasmic staining and accumulation in bright intracellular particles, 1–6 µm in size. The number of cells positive for cleaved IL-1β was significantly (p = 0.001) higher in the 5% BFSE treated THP-1 macrophage cells with 53.7% positive compared with 11.2% for the air treated control cells (Fig. 3E).

### BFSE alters secretion of pro-inflammatory chemokines

**Figure 4:**
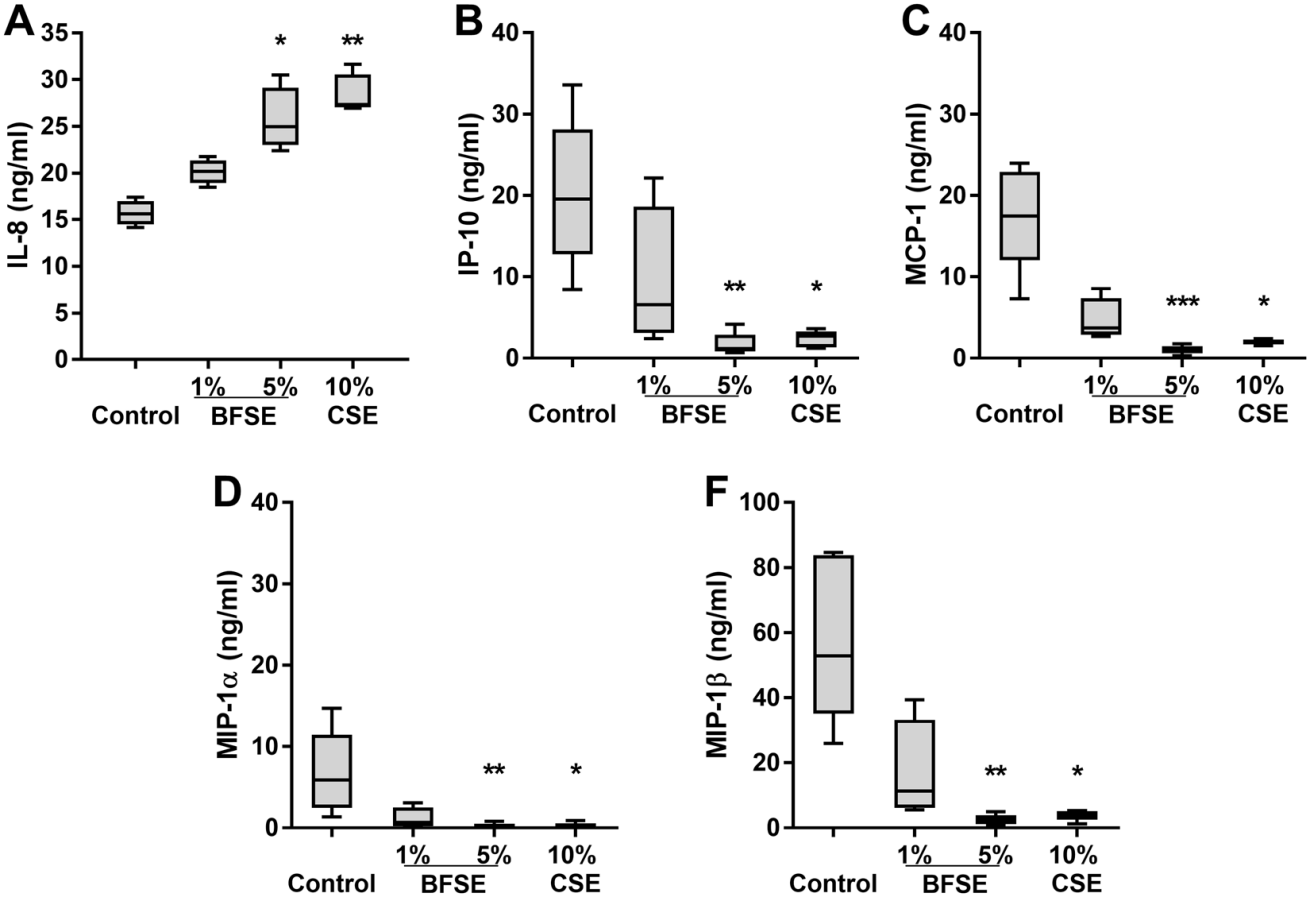
BFSE alters THP-1 macrophage chemokine secretion. THP-1 macrophage cells were exposure to air control or 1% or 5% BFSE or 10% CSE for 24 h. Supernatants were collected and assessed for IL-8 (**A**), IP-10 (**B**), MCP-1 (**C**), MIP-1α (**D**) and MIP-1β (**F**) by chemokine bead array and analysed by flow cytometry (n = 5). *p < 0.05 **p < 0.01 ***p < 0.001.

We investigated the impact of BFSE exposure on THP-1 macrophage secretion of pro-inflammatory chemokines. Secretion of IL-8 was significantly increased from 15.7 ng/mL to 25.9 ng/mL (p < 0.05) and 28.6 ng/mL (p < 0.01) after 24 h exposure to 5% BFSE and 10% CSE, respectively (Fig. 4). Conversely, secretion of other pro-inflammatory chemokines: IP-10, MCP-1, MIP1α and MIP-1β were all significantly decreased in the supernatant of cells treated with 5% BFSE and 10% CSE (Fig. 4).

### BFSE decreases macrophage viability

#### LDH release

**Figure 5:**
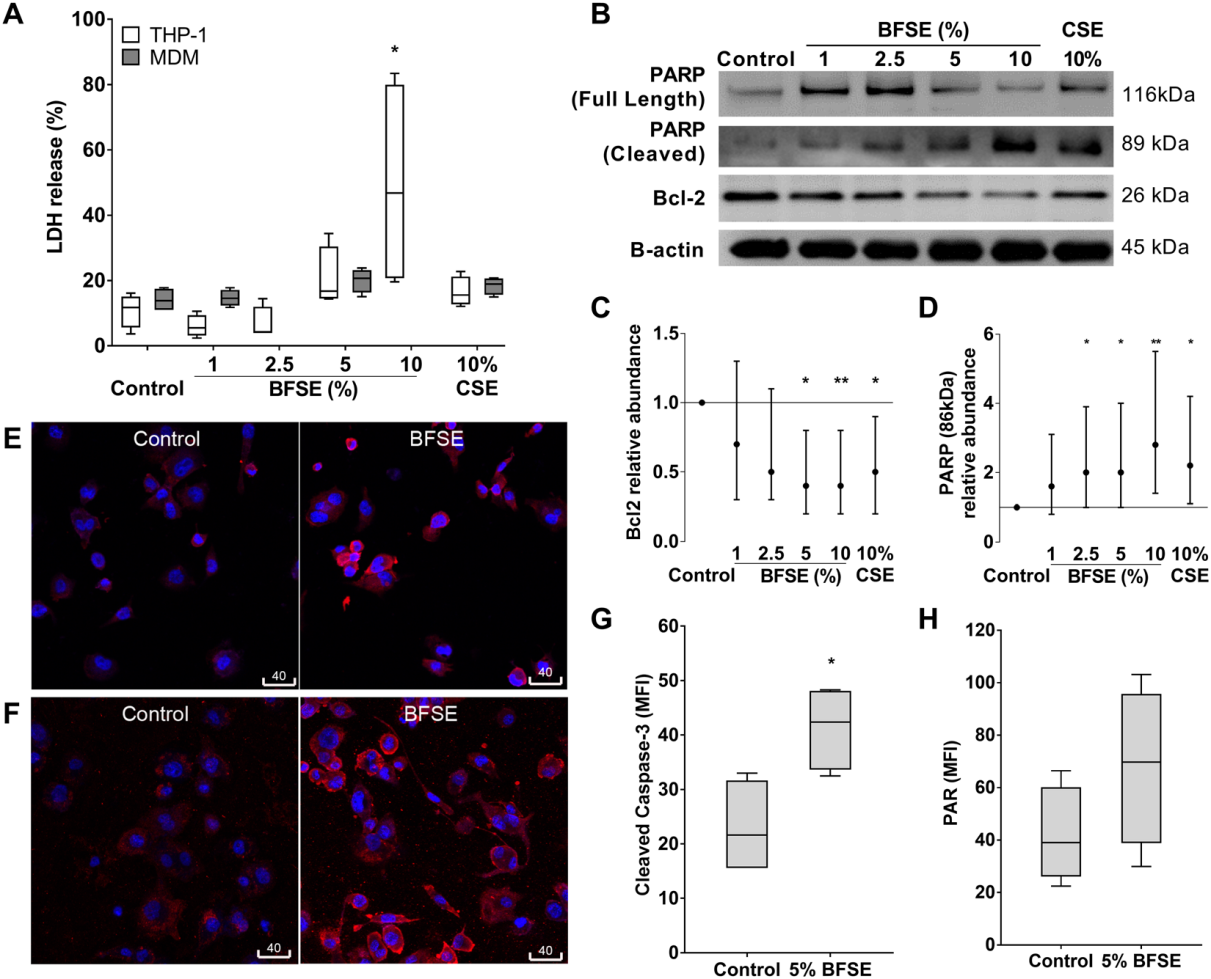
BFSE decreases macrophage viability. THP-1 macrophage, or MDM cells, were exposure to air control or 1%, 2.5%, 5%, 10% BFSE or 10% CSE for 24 h. LDH from THP-1 macrophage or MDM cells was measured in supernatants after 24 h (n = 4) (**A**). THP-1 macrophage expression of Bcl-2 and PARP was assessed by western blot (**B**), band densitometry analysis of Bcl-2 (**C**) and PARP (**D**) was performed, data is a representation of three independent experiments, and was baselined to the air treated control sample, and normalized to β-actin expression. Error bars represent 95% confidence intervals. Representative confocal images of active caspase-3 (**E**) and PAR (**F**), in THP-1 macrophage with quantitative MFI measurement of active caspase-3 (**G**) and PAR (**H**). (n = 4) *p < 0.05, **p < 0.01.

LDH released from THP-1 macrophages increased in a dose-dependent manner after 24 h exposure to 1–10% BFSE, with mean LDH release in response to 10% BFSE increasing from 10.8% to 49.1% compared to air-treated control cells (p = 0.029). THP-1 macrophages and MDM cells treated with 5% BFSE exhibited LDH release that was consistent with exposure to 10% CSE but not significantly different from air treated control (20.5% and 16.5% vs 10.8% for THP-1, 19.3% and 17.8% vs 14.1% for MDM, respectively) (Fig. 5A).

#### Bcl-2, PARP and active caspase-3

Western blot analysis of protein from THP-1 macrophages exposed to 1–10% BFSE exhibited a dose-dependent decrease in the anti-apoptotic Bcl-2 and a concomitant increase in PARP cleavage compared to air treated control (Fig. 5B). The increases were confirmed using densitometry analysis (Fig. 5C,D).

Immunostaining for pro-apoptotic active caspase-3 was detected in the air treated control THP-1 macrophages and increased in cells exposed to 5% BFSE for 24 h (Fig. 5E). Image analysis of intracellular active caspase-3 revealed a significant (p = 0.021) increase in response to 5% BFSE compared to air treated control, MFI: 41.3 vs 23.0 (Fig. 5G). Immunostaining for Poly (ADP-ribose) (PAR, Fig. 5F), a polymer formed by active PARP, and an indicator of DNA damage and inducer of pro-apoptotic factor release from mitochondria^26^ was non-significantly (p = 0.195) increased (68.1 vs 41.7), in 5% BFSE vs control (Fig. 5H).

#### 7AAD and Annexin V

Treatment with BFSE or CSE did not cause a significant decrease in the percentage of apoptotic/necrotic cells (which could potentially account for a decrease in phagocytosis or receptor expression), assessed by flow cytometric analysis of annexin V and 7AAD, compared to untreated cells (Supplementary Fig. 3) The percentage of total events falling in the macrophage ‘Macs” gate were not significantly affected by BFSE or CSE treatment.

## Discussion

This study investigated changes to macrophage function and viability after exposure to smoke extract from common Australian flora. Previous studies have largely focused on the effects of cigarette smoke and fractions of particulate matter. However, the smoke produced from combustion of plant material also contains potentially damaging components including aldehydes, acrolein, and carbon monoxide^15^. To investigate the effects of BFSE we adapted our current methods used for the investigation of cigarette smoke^27^. The concentrations of BFSE were extrapolated from the previous findings of our group and others showing that CSE at 10% is sufficient to elicit an inflammatory response and impair macrophage function^22,27,28^. This concentration has been shown to be equivalent to smoking 0.5–2 packs of cigarettes per day^29^.

Macrophage efferocytic clearance of apoptotic cells in the airway is important to prevent secondary necrosis of the uncleared apoptotic material and a resultant progression of inflammation^19,25^. We have shown significantly reduced efferocytosis in the airways of patients with a range of chronic lung diseases including COPD and severe asthma^18,19,22,23,30^. In the present study, the capacity of THP-1 macrophages and MDM to efferocytose apoptotic bronchial epithelial cells was significantly impaired after exposure to BFSE, findings consistent with our previously reported data following CSE and E-cigarette exposure^31^, and with a report that acrolein, a by-product of burning wood or cigarettes, reduced the ability of macrophages to engulf erythrocytes^32^. Our findings may shed further light on the reported increases in COPD and asthma patient admissions associated with bushfire events^8,9^. As COPD patients and severe asthmatics already have compromised alveolar macrophage phagocytic function and an increased rate of lung epithelial cell apoptosis, the additional bombardment by bushfire smoke would likely compound their respiratory morbidities.

Chronic bacterial airway colonisation with potentially pathogenic bacteria including NTHi has been linked to the pathogenesis of several chronic lung diseases including COPD and severe asthma, with NTHi accounting for up to half of all isolates in COPD^33^. Efficient phagocytosis of this bacteria by alveolar macrophages is thus important to reduce bacterial colonisation, limit inflammation and to prevent exacerbations (in COPD, up to 80% of exacerbations are associated with this bacteria^33^. In the present study, BFSE significantly suppressed the capacity of THP-1 or MDM macrophages to phagocytose NTHi, again consistent with our findings with CSE and in patients with chronic lung diseases where the defective clearance was correlated with increased markers of inflammation, and potentiation of bacterial colonisation of the lower airways.

Data from BFSE-exposed THP-1 macrophages was consistent with our previous data from both CSE-exposed THP-1 macrophage cells^27^ and primary alveolar macrophages from heathy smokers and patients with COPD^18,19,34^. Nevertheless we also performed experiments using primary monocyte derived macrophages (MDM). The additional experiments with MDM showed higher initial levels of phagocytosis and more marked inhibition with BFSE and CSE, findings that are consistent with our previous studies. Consistent with the changes in percentage of cells phagocytosing, we also noted a decrease in MFI, suggesting that CSE and BFSE also affect the amount of prey being taken up. The level of inhibition induced by BFSE and CSE is likely to be physiologically relevant, as our previous studies in smoke-exposed mice treated with macrophage-targeted therapies showed an improved macrophage phagocytic capacity that significantly correlated with a reduction in inflammation.

We also previously found that the deficiency appeared to be specific for apoptotic cells or bacteria, as tests carried out in parallel using carboxylate modified polystyrene microbeads revealed no significant difference between COPD patients and control subjects^18,30^. One reason for this deficiency may be defective recognition of the phagocytic target in response to cigarette or bushfire smoke, a concept that is supported by the findings in the present study and in our previous studies of cigarette smoke exposure^22,34,35^. The interaction between macrophages and phagocytic targets is mediated by a variety of macrophage membrane-associated proteins. We therefore applied primary MDM to investigate the effects of BFSE on several recognition molecules that can be used in both phagocytosis of bacteria and efferocytosis. BFSE, consistent with the effects of CSE, significantly decreased the percentage of MDM expressing thrombospondin receptor (CD36), class A scavenger receptors (SR-As), mannose receptor (CD206) and TLR-2 but not TLR-4. Ligation of the hyaluronan receptor (CD44) is also important for efficient clearing of excess hyaluronan and apoptotic cells that may otherwise contribute to tissue damage in COPD. We noted a significant suppression of CD44 expression by MDM in response to BFSE, consistent with our previous report of decreased expression of CD44 on alveolar macrophages from smokers with or without COPD compared with healthy never-smoker controls^22^. We also noted a reduction in the MFI of CD36, TLR2, TLR4 and CD44, indicating that the both the percentage of cells expressing the receptors and the amount of receptors being expressed are being reduced. This data indicates that cells that are still positive for the receptors after exposure to smoke are expressing less receptors. Taken together, this data suggests that the suppressed recognition of phagocytic targets, whether apoptotic cells or bacteria, in response to BFSE or CSE may one mechanism for the defective phagocytic clearance, contributing to bacterial persistence and increased inflammatory response. A potential limitation of our study is the effect of variable numbers of non-viable macrophages in the macrophage gate on receptor expression. We however consider this very unlikely as we used an identical protocol across multiple experiments comparing control and treatment data, and our flow cytometry gating ensured that greater than 93% of viable cells were included in the analyses.

A further pro-inflammatory effect of BFSE on THP-1 macrophages was an increase in active caspase-1 and IL-1β, two cleavage products downstream of inflammasome assembly. These data are consistent with reports that aldehydes and acrolein, components in both wood and cigarette smoke^15^, induced secretion of IL-8 and other inflammatory mediators from macrophages^28^. Interestingly, we noted a decrease in THP-1 secretion of pro-inflammatory mediators MCP-1, IP-10, MIP-1α and MIP-1β following exposure to BFSE or CSE. This was surprising given the increases in intracellular IL-1β; but consistent with the findings of many other groups. For example, decreased secretion of TNFα, IL-1β, IL-6, MIP-1α, MIP-1β, IP-10 and MCP-1 was reported in macrophages exposed to cigarette smoke or in cultured alveolar macrophages from smokers compared with non-smokers^28,36–45^.

Maintaining macrophage viability in the airway is important to ensure effective phagocytic function. Our dose response studies confirmed that 10% BFSE had the most cytotoxic effects on THP-1 macrophages while even 5% BFSE reduced macrophage viability to a similar extent as 10% CSE. The reduction in macrophage viability in response to 5% BFSE was accompanied by a decrease in the anti-apoptotic protein Bcl-2 and increased activation of caspase-3, suggesting that initiation of apoptosis is at least in part responsible for the decrease in macrophage survival. These findings are consistent with those of Jalava *et al*.^46^ who found an equivalent amount of apoptosis, up to 17%, in mouse macrophage RAW264.7 cells after 24 hours of exposure to particulate matter from woodland fires.

Taken together, our data shows that bushfire smoke exposure can impair macrophage function and viability and increase production of pro-inflammatory mediators, to a similar extent as CSE, and highlights the need for further research into the harmful effects of bushfire smoke, with particular relevance to patients with pre-existing lung disease.

## Methods

### Preparation of smoke extracts

#### Cigarette smoke extract (CSE)

As a known affecter of macrophage function, a single batch of 100% stock cigarette smoke extract, which was used throughout the study as a reference for the impact of bushfire smoke, was prepared as previously reported^22^. Briefly, the smoke from three 1R5F research-reference filtered cigarettes containing 1.67 mg of tar and 0.16 mg of nicotine (The Tobacco Research Institute, University of Kentucky, Lexington, KY) was bubbled through 30 mL RPMI 1640 medium supplemented with 10% foetal bovine serum (FBS), penicillin/gentamicin (all Thermo Fisher Scientific, MA, USA) at a speed of 5 min per cigarette using a vacuum pump. The pH was adjusted to neutrality and aliquots of the cigarette smoke extract were stored at −80 °C.

#### Bushfire smoke extract (BFSE)

Equal weights of the following species (indigenous or common introductions to the bushfire prone region of the Adelaide Hills, South Australia) were combined: *Acacia baileyana* (Cootamundra wattle) leaves and stems, *Acacia melanoxylon* (blackwood) leaves and stems, *Acacia vestita* (weeping acacia) leaves and stems, *Eucalyptus camaldulensis* (river red gum) leaves and *Eucalyptus globulus* (blue gum) leaves.

Each species was blended separately using a CG2B spice grinder (Breville, Sydney, NSW, Australia). The blended material was weighed and equal portions were mixed together, half of the mixture was frozen immediately at −80 °C (‘wet’) while the other half was dehydrated using a DT5600 food dehydrator (Sunbeam, Botany, NSW, Australia; ‘dried’) for 4 h, at setting two (approximately 55 °C) then stored in a desiccator.

To prepare the 100% stock smoke extract: smoke from 2 g of ignited foliage (1.5 g dried plus 0.5 g wet), a mass proportionate to the cigarette mass used for 100% CSE, was bubbled through 20 mL of HEPES buffered saline solution using a vacuum pump taking 15 min to burn all material. The pH was measured and adjusted to neutrality if required and aliquots were stored at −80 °C. A control solution of HEPES buffered salt solution exposed to bubbled air for 15 min and stored at −80 °C was prepared in parallel. For initial optimisation experiments, concentrations of 1–10% were investigated, and a final concentration of 5% used in all experiments. 100% BFSE was diluted into RPMI 1640 medium with 10% FBS, penicillin/gentamicin.

### Preparation of Cell Lines

The monocytic cell line, THP-1 (American Type Culture Collection, Manassas, VA, USA) was maintained at 37 °C/5% CO_2_ in RPMI 1640 medium supplemented with 2 mM L-glutamine, 10% FBS, penicillin/gentamicin and 0.05 mM ß-mercaptoethanol (Sigma-Aldrich, MO, USA). Differentiation into macrophages was facilitated by seeding at a density of 5 × 10^5^ cells/mL in culture medium supplemented with 50 nM phorbol 12-myristate 13-acetate (PMA; Sigma-Aldrich) for 72 h as previously described^47^.

The 16HBE14o- airway epithelial cell line was a generous gift from Dr Dieter C. Gruenert (University of California, San Francisco, USA). 16HBE14o- cells were maintained in MEM medium supplemented with 2 mM L-glutamine, 10% FBS, penicillin/gentamicin under humidified 37 °C/5% CO_2_ conditions. Cell culture materials were from Thermo Fisher Scientific unless stated otherwise.

### Preparation of Monocyte Derived Macrophage (MDM)

Adult controls were recruited from our volunteer database, were non-smokers and had no history of respiratory or allergic disease. Patients were invited to participate in the study, and fully informed consent was obtained. The study protocol was approved by the Royal Adelaide Hospital Research Committee (#020811d). All research was performed in accordance with relevant guidelines and regulations. Monocytes were isolated from whole blood collected in Lithium-Heparin tubes (Greiner Bio One, Austria). Blood was diluted with 2 volumes of RPMI 1640 medium without additives and layered over Lymphoprep^TM^ (STEMCELL Technologies, BC, Canada). Peripheral blood mononuclear cells (PBMC) were isolated as per manufactures instructions. PBMC were seeded into plates at 1.4 × 10^6^/mL in RPMI 1640 medium without additives at 37 °C/5% CO_2_ for 60–90 min to allow monocytes to adhere. Macrophage were derived from monocytes cultured in RPMI 1640 medium supplemented with 2 mM L-glutamine, 10% FBS, penicillin/gentamicin and 2 ng/mL granulocyte-macrophage colony-stimulating factor (GM-CSF, Sigma-Aldrich) for 12 days with full media changes at 4 and 8 days.

### Phagocytosis Assays

Phagocytosis of apoptotic 16HBE14o-bronchial epithelial cells or non-typeable *H. influenzae* (NTHi) by differentiated THP-1 macrophages or MDM exposed to smoke extracts or control media was performed as previously reported^48^. Briefly, NTHi stained with Sytox Green and 16HBE14o- stained with Sytox Orange from Thermo Fisher Scientific were incubated with MDM or THP-1 macrophage cells, at a 100:1 and 5:1 ratio respectively, for 90 min before being lifted and analysed by flow cytometry on a FACSCanto II (BD Biosciences, San Diego, USA). Gating procedures have been previously published^18,27,31,35^.

### Flow cytometry of cell surface markers

MDM cells were incubated in ice cold PBS for 15 min before lifting with a bulb pipette. Cells were washed with isoflow (BD Biosciences) containing 0.5% bovine serum albumin (Sigma-Aldrich) and pelleted. Cells were incubated with 2 µL of conjugated primary antibodies to SR-A1 (#FAB2708A; APC; R&D Systems, MN, USA); TLR-2 (#12-9024; PE) or TLR-4 (#17-9917; APC; eBiosciences, CA, USA); CD36 (#IM0766U; FITC) or CD206 (#IM2741; PE; Beckman Coulter, IN, USA); or CD44 (#555478; FITC; BD Bioscience) for 10 min in the dark, and washed. All antibodies were titrated to determine optimal antibody concentration to exclude non-specific binding. Cells were stained with isotype controls to exclude autofluorescence/non-specific binding for quadrant marker placement to include 98% of the gated cell population i.e., less than 2% positive staining^35^.

Fifty thousand events were collected and cell surface receptors analysed, using FACS DIVA 7.0 and expressed as % positive of cells expressing the marker. To further assess the amount of receptor being assessed and potential changes in response to BFSE and CSE, we also recorded MFI of receptor expression. Gating procedures have been previously published^18,27,31,35^.

### Immunofluorescence and confocal microscopy

The apoptotic markers: cleaved caspase-3 and poly (ADP-ribose) (PAR, a polymer formed by active PARP (poly (ADP-ribose) polymerase)) and the inflammatory markers cleaved caspase- 1 and total/cleaved IL-1β were assessed in THP-1 macrophages exposed to 5% BFSE using a method described earlier for CSE exposure^27^. Briefly, cells were fixed with 2.5% formalin in phosphate-buffered saline (PBS), permeabilized with 0.1% Triton X-100 (Sigma-Aldrich) in PBS, pre-blocked with serum-free protein blocker (Dako, Glostrup, Denmark), incubated overnight at 4 °C with primary antibodies and 1 h at room temperature with secondary antibodies. Primary antibodies were rabbit polyclonal anti-cleaved caspase-3 (1/40, R&D Systems), mouse monoclonal anti-PAR (1/20, Enzo Life Sciences, NY, USA), and rabbit polyclonal anti-IL-1β (1/30, H-153) goat polyclonal anti-cleaved caspase-1 (1/20, h297) and goat polyclonal anti-cleaved IL-1β (1/20, h117) (all Santa Cruz Biotechnology). All secondary antibodies were donkey IgG F(ab’)_2_ fragments with Alexa Fluor (AF) conjugates from Jackson ImmunoResearch (West Grove, PA, USA); anti-rabbit IgG (AF594 or AF647), anti-goat IgG (AF488), and anti-mouse IgG (AF647). Images were captured on a LSM700 confocal microscope (Carl Zeiss Australia, NSW, Australia). For quantitative analysis, 10 serial images at a 20× objective were captured from each well of an 8-well chamber slide in a blinded manner by focusing on the DAPI channel. Measurement of mean fluorescence intensity (MFI) or percentage of brightly fluorescent cells was determined by ImageJ morphometric software (NIH, Bethesda, MA, USA).

### Cytometric Bead Array (CBA)

Supernatant from THP-1 macrophage cells exposed to smoke extracts or air control for 24 h were assessed with a human inflammatory chemokine CBA kit (BD Biosciences), as per manufacturer instructions. Chemokines: IL-8, MCP-1, IP-10, MIP-1α and MIP-1β were measured on a FACSCanto II and analysed with FCAP Array software (BD Biosciences).

### Assessment of macrophage viability

LDH, an enzyme released from cells with compromised membranes was measured in supernatant from THP-1 macrophage or MDM as instructed by a cytotoxicity detection kit (Roche; Mannheim, Germany). Briefly, supernatants were centrifuge at 500 × g to remove cell debris then stored at −80 °C prior to analysis. Supernatant were added to a 96-well plate in triplicate followed by reaction mixture then incubated at room temperature in the dark for 10 min. The assay reaction was neutralised with the provided stop solution and absorbance measured at 490 nm, with a 600 nm reference wavelength, on an Epoch microplate spectrophotometer (Bio-Tek; VT, USA).

### Western Analysis

For western blot analysis of the anti-apoptotic B-cell lymphoma 2 (Bcl-2) and poly-ADP ribose polymerase (PARP) cleavage, THP-1 cells were lysed using M-PER mammalian cell protein lysis reagent with Halt® protease inhibitor cocktail (Thermo Fisher Scientific). Protein samples were quantified using Bio-Rad (CA, USA) DC protein assay; 10 µg of protein (was electrophoresed on 4–12% gradient Bis-Tris gels before being transferred to nitrocellulose membrane. Membranes were blocked in 5% diploma skim milk (Fonterra, NZ) or 5% bovine serum albumin before probing with primary antibodies and corresponding horseradish peroxidase-conjugated secondary antibodies (R&D Systems). Band detection was performed using ECL Prime chemiluminescent substrate (GE Healthcare, Buckinghamshire, UK), on a LAS-4000 instrument with Multigauge software for densitometry analysis (FugiFilm, Tokyo, Japan). Primary antibodies were: PARP and Bcl-2 (rabbit polyclonal, Santa Cruz Biotechnology, CA, USA), with β-actin (mouse monoclonal, Sigma-Aldrich) used for loading correction during band analyses.

### Flow cytometric analysis of 7AAD and Annexin V staining

Macrophages exposed to air control, 1% BFSE, 5% BFSE or 10% CSE for 24 h were assessed by flow cytometry for cell viability using 7AAD and Annexin V as published^49,50^.

### Statistical analysis

Data were analysed using SPSS software (SPSS Inc. IBM Chicago, USA). Results are reported as min, max and median (q1, q3) unless otherwise indicated. Analysis was performed using the non-parametric Friedman test, with Wilcoxon signed rank tests for post-hoc pairwise comparisons. A value of p < 0.05 was considered statistically significant. Densitometry analysis of protein bands was performed using Multi Gauge software (V3.1 Fugifilm, Tokyo, Japan). Density scores were analysed by a gamma (log link) mixed model regression to allow for correlated treatment responses within each culture. Results are normalized to both β-actin and the air control; and presented as relative protein expression. Analysis was performed using R statistical software (release 3.2.3), and library lme4 (v 1.1–12)^50,51^.

## Electronic supplementary material

Supplementary figures
