# Supplementary material for: Bushfire smoke is pro-inflammatory and suppresses macrophage phagocytic function
Source: Sci Rep. 2018 Sep 7;8:13424. doi: 10.1038/s41598-018-31459-6 (PMC6128914; doi:10.1038/s41598-018-31459-6)
Supplement: Supplementary file 1 — Supplementary figures [file 41598_2018_31459_MOESM1_ESM.docx]

**Supplementary data: Bushfire smoke is pro-inflammatory and suppresses macrophage phagocytic function.**

Rhys Hamon^1, 2^, Hai B Tran^1, 2^, Eugene Roscioli^1, 2^, Miranda Ween^1, 2^, Hubertus Jersmann^1, 2^, Sandra Hodge^1, 2^

*^1^Chronic Inflammatory Lung Disease Research Laboratory, Department of Thoracic Medicine, Royal Adelaide Hospital, Adelaide, Australia, ^2^Department of Medicine, University of Adelaide, Adelaide, Australia*

**Methods**

**Annexin V and 7-AAD (7-Aminoactinomycin D) Viability analysis**

Macrophage cells exposed to air control, 1% BFSE, 5% BFSE or 10% CSE for 24 h were lifted in cold 1x PBS and pelleted. Cells were washed and resuspended in Annexin binding buffer (ABB; 10 mM HEPES, 150 mM NaCl, 5 mM KCl, 1.8 mM CaCl_2_, 1 mM MgCl_2_) before being stained with 25ug/mL 7-AAD (Sigma-Aldrich, Castle Hill, NSW, Australia) and Annexin V-FITC (BD Bioscience) as previously described^1^. Stained cells were accessed by flow cytometry using a FACScanto II (BD Bioscience) equipped with FACSDiva.

**Results**


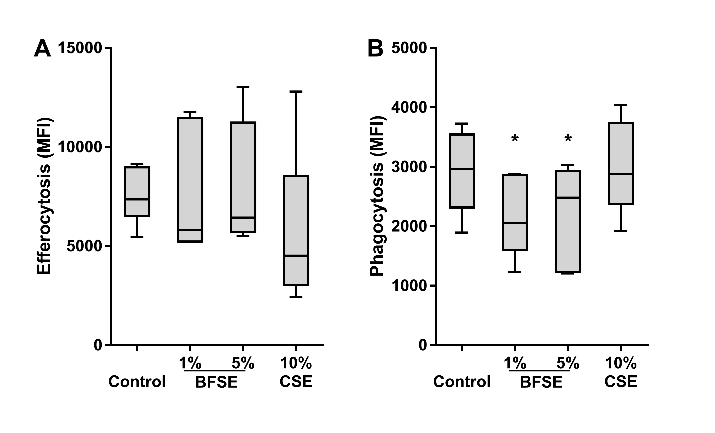


**Figure S1. BFSE decreases the rate of phagocytosis of NTHi by MDMs**

MDM cells were exposed to air control, 1% or 5% BFSE, or 10% CSE for 24 h were cultured with apoptotic epithelial cells and NTHi. (A) Efferocytosis of apoptotic epithelial cells and (B) phagocytosis of NTHi by MDM were assessed by flow cytometry for changes in mean fluorescent intensity (MFI) to determine any changes in the number of targets internalisation (MDM n=6). *p<0.05.


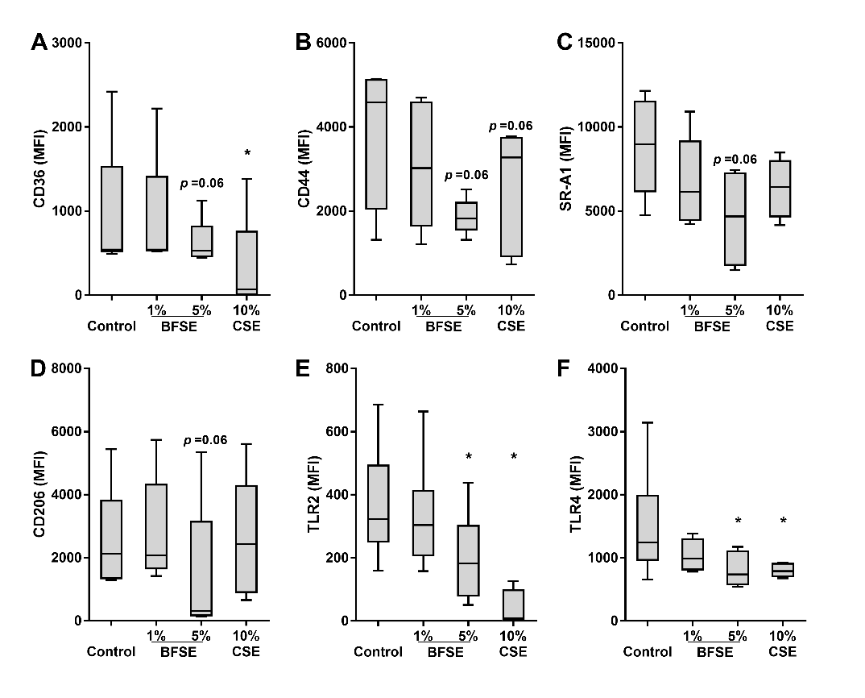


**Figure S2. BFSE alters surface expression of phagocytic recognition receptors**

MDM were exposure to air control, 1% or 5% BFSE or 10% CSE for 24 h. Changes in cell surface expression of phagocytic receptors: CD36 (A), CD44 (B), SR-A1 (C), CD206 (D), TLR-2 (E) and TLR-4 (F) were detected by immunofluorescence on a FACScanto II flow cytometer. Receptor expression is presented as MFI above negative control MFI. (n=5) *p<0.05.


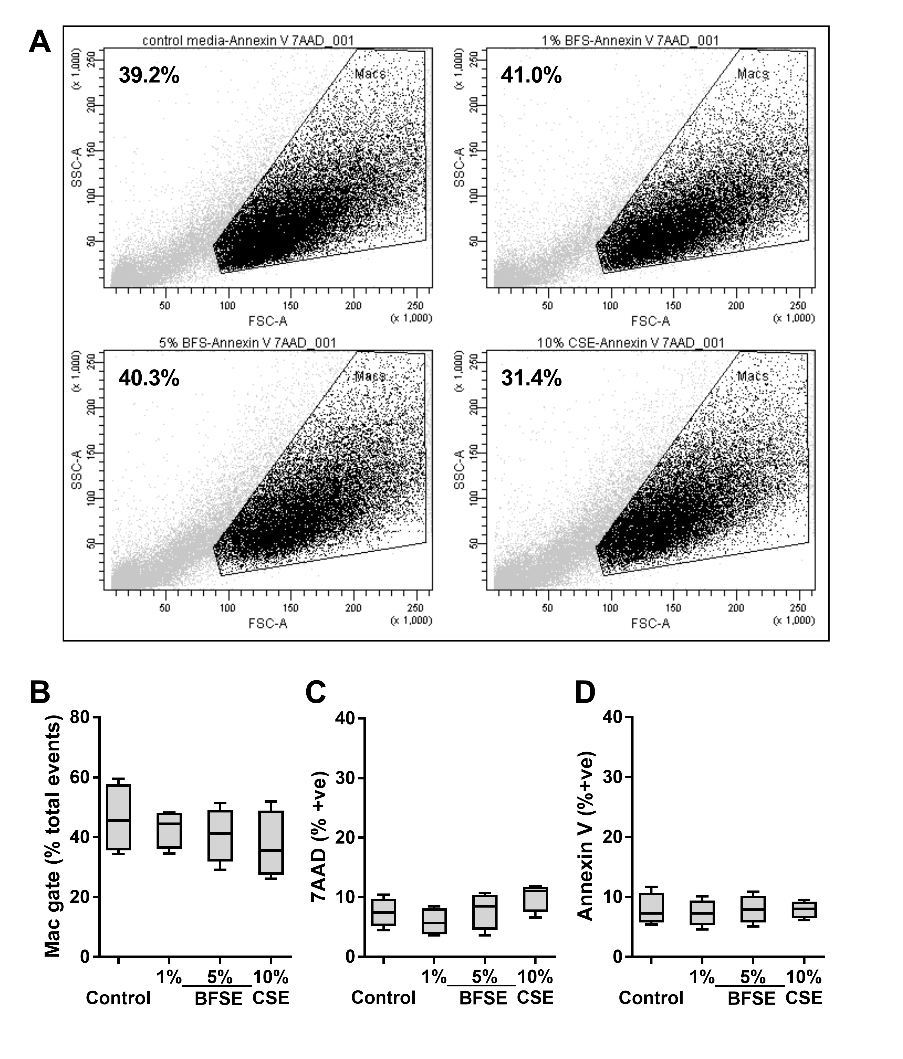


**Figure S3. BFSE treatment did not alter viability of gated macrophage**

Macrophage cells exposed to air control, 1% BFSE, 5% BFSE or 10% CSE for 24 h were assessed by flow cytometry for cell viability using 7AAD and Annexin V. (A) Representative dot plots of gated macrophage cell populations used for analysis. (B) The percentage of total events falling in the macrophage ‘Macs” gate were not significantly affected by BFSE or CSE treatment. The percentage of 7-AAD positive (C) or Annexin V (D) positive cells were not changed after exposure to BFSE or CSE, compared to air control n=4).

References

1 Hodge, G. L., Flower, R. & Han, P. Optimal storage conditions for preserving granulocyte viability as monitored by Annexin V binding in whole blood. *Journal of immunological methods* **225**, 27-38 (1999).
